# Shoreline development influences common loon territory selection but not extent of nest defense

**DOI:** 10.1093/beheco/arag133

**Published:** 2026-10-17

**Authors:** Emily Renkey, Katie A Adler, Daniel T Blumstein, Walter H Piper

**Affiliations:** Department of Ecology and Evolutionary Biology, 621 Charles E Young Drive South, University of California, Los Angeles, CA 90095-1606, United States; Department of Ecology and Evolutionary Biology, 621 Charles E Young Drive South, University of California, Los Angeles, CA 90095-1606, United States; Department of Ecology and Evolutionary Biology, 621 Charles E Young Drive South, University of California, Los Angeles, CA 90095-1606, United States; Schmid College of Science and Technology, Chapman University, Orange, CA 92866, United States

**Keywords:** common loon, *Gavia immer*, flight initiation distance, natal habitat preference induction, human impacts, shoreline development

## Abstract

Shoreline development of temperate lakes has increased steadily in recent decades, putting pressure on animal populations that live in lacustrine habitats. Common loons (*Gavia immer*) nest along lakeshores, and shoreline development has been identified as a likely cause of population decline in this species. Taking advantage of a marked loon population from a long-term study in northern Wisconsin, USA between 1992 and 2023, we investigated impacts of shoreline development and human disturbance on territory choice and nest defense behavior of breeding loons. Loons settled on territories that resembled their natal lakes in terms of shoreline development, an indication of natal habitat imprinting. Shoreline development did not affect the distance at which loons flushed from their nest at the approach of humans. However, nesting loons were less likely to flush from the nest late in the incubation period, suggesting a tendency for loons to defend nests according to likely fitness benefits. Loons' tendency to breed on lakes similar in human activity to their natal lake might reflect their effort to seek out lakes of familiar fish species composition rather than human activity. Natal habitat imprinting of this kind illustrates the potential of anthropogenic factors to affect behavior and, in turn, population dynamics.

## Teaser

The level of shoreline development on lakes affects where common loons breed. In our study, loons selected breeding territories with a level of shoreline development that matched the lake where they were hatched. Shoreline development did not affect nest defense during incubation, and loons defended their nests more strongly when their eggs were close to hatching.

## Introduction

Shoreline development has complex impacts on a wide range of lake-dwelling animals. Past studies have linked shoreline development to reductions in the abundance of invertebrates (Twardochleb and Olden 2016) and amphibians (Woodford and Meyer 2003), reduced growth rates in fish species (Schindler et al. 2000), and altered avian community composition (Lindsay et al. 2002). Lakes lacking residential development are becoming increasingly rare. Habitat modification and human presence have also been shown to change nesting behavior in fishes (Lawson et al. 2011) but these behavioral changes across species are understudied, despite their potential to have important implications for fitness outcomes (Uchida and Blumstein 2021).

Common loons (*Gavia immer*, hereafter loons) are migratory waterbirds that are particularly well suited to study these behavioral modifications. Loons are found primarily in the coniferous zone of North America during their breeding season and have high site fidelity to their breeding territory (Paruk et al. 2021). Additionally, loons use shoreline and littoral zones on freshwater lakes for nesting and chick-rearing, making their nests and young vulnerable to human activity. Birds that incubate their eggs must respond adaptively to threats to their nests and themselves. With loon populations declining in many regions, including at the southern end of their breeding range (Piper et al. 2020; Saunders et al. 2021), understanding how loons respond to human development is crucial for management. Indeed, development and recreation along shorelines have been shown to increase rates of chick mortality and nest abandonment (Caron and Robinson 1994; Burgess and Meyer 2008; Spilman et al. 2014), highlighting a need for further study of the full extent of development's impact on loon behavior and fitness.

Loon chicks fledge from their natal territory in the fall, migrating to the coasts of North America for winter (Evers 2004). Loons exhibit short-range natal dispersal, returning to the region they fledged and settling on a nearby territory to breed between 2 and 4 yr of age. Since loons, especially males, return to the same region they were born, identification across successive life stages is possible (Piper et al. 2012). Young adults use at least 2 strategies for selecting territories on which to settle. The first is Natal Habitat Preference Induction (NHPI), where loons settle on lakes that resemble their natal lake in area and pH (Piper et al. 2013). Second, loons prospect for territories with chicks and attempt to evict owners from those territories, usually in the following year (Piper et al. 2006). Habitat selection has profound consequences for individual fitness, as natal habitats can influence reproductive success and future distribution of individuals across a landscape (Fletcher et al. 2015). While it is plausible that loons also use the level of human activity as a cue to guide territory settlement, no study has yet looked at this possibility.

Once loons settle on a breeding territory, they spend 3 to 4 mo there to incubate their eggs and rear their chicks. Hence, the conditions of the lake on which a breeding territory is established can greatly impact loon survival and breeding success. Human activity on lakes has been directly linked to both individual loon mortality and population declines, with poisoning from lead fishing tackle identified as the leading cause of mortality in adult common loons (Grade et al. 2018). Less is known, however, about indirect anthropogenic impacts on loon behavior, especially during the breeding season.

Flight initiation distance (FID), or the distance at which an animal flees in response to an approaching threat, is often used by behavioral ecologists to determine an animal's perception of risk (Cooper and Blumstein 2015), including during incubation. FID can be seen as a cost-benefit analysis, where an animal will delay fleeing and continue their activities, weighing the shifting costs as the predator approaches (Ydenberg and Dill 1986). The decision to remain on the nest decreases harm to the eggs but increases the potential survival risk to the parent (Montgomerie and Weatherhead 1988). This cost-benefit curve changes throughout the course of incubation, with parents being more likely to remain on the nest as the value of their offspring increases. Birds from urban habitats generally have shorter FIDs compared with their conspecifics in areas with low human densities, indicating that increased human presence influences their risk-taking behavior (Møller 2015). Past studies have examined differences in individual-level FID of reptiles and mammals in varying disturbance conditions (Li et al. 2011; McGowan et al. 2014). However, very few avian studies have examined FID in known individuals (Livezey et al. 2016). Housing density near shorelines of northern Wisconsin lakes has increased by more than 600% in the past 100 yr, a trend that is expected to continue (Perales and Vander Zanden 2023). With loon populations predicted to decline in coming decades, we must better understand how human activity in their breeding habitats influences their behavior.

We sought to determine: (I) how residential shoreline development influences territory selection in first-time breeding loons when a variety of territories are available, and (II) whether the density of shoreline development affects risk assessment in incubating loons. We predicted that juvenile loons would settle on a breeding territory with a similar level of dock density and developed land cover to that of their natal territory because individuals from a particular type of habitat might perform better if they recruit to the same type of habitat (Davis and Stamps 2004). We further predicted that loons on lakes with a higher dock density would assess lower risk from a canoe approaching and flush into the water at a shorter distance compared with loons on lakes with lower dock density. We also predicted that loons would tolerate closer approaches (ie, show greater nest defense) when eggs are close to hatching because of greater past investment as well as the time and energy that it would require to get to the same point again in the future (Weatherhead 1979; Montgomerie and Weatherhead 1988).

## Materials and methods

### The study system

We studied loons in a roughly 1,700 km^2^ region in north-central Wisconsin, USA (geographic center at 45.77733° N, −89.58738° W) that contains about 200 glacial lakes and is bordered by upland forest, marsh, and sphagnum bog. The region experiences a high degree of anthropogenic activity in the form of angling and boating, but the degree of shoreline development and recreational use varies substantially from one lake to another. This activity varies seasonally, with the increase in human use aligning with the breeding season for loons from mid-May through mid-August.

Breeding common loon territories consist of entire small lakes or protected bays on large lakes. In this region, loon territories comprise either entire small lakes (*N* = 130; mean size ± SD = 42 ± 34 ha), or mapped portions of large lakes (*N* = 54 territories on 24 different lakes; mean size ± SD = 387 ± 403 ha). Pairs nest chiefly on islands, bogs, or emergent vegetation within marshes. The male and female jointly incubate a 1-or-2 egg clutch which hatch after 28 d. Parents feed and protect the chicks until they fledge at about 11 wk (Paruk et al. 2021).

### Field methodology

We captured adult loons and chicks at night from a small motorboat using a spotlight and a large fishing net (Evers 1993). We then banded adults and chicks that were at least 4 wk old with one U.S. Geological Survey metal band and 3 colored plastic bands (Gravoglas 2-Plex: GravoTech, Inc., Duluth, GA, USA) in a unique combination to allow for individual identification. About 40% of all male chicks and 15% of all female chicks that we banded returned 2 to 4 yr later as adults to look for breeding territories (Piper et al. 2012, 2015), and roughly two-thirds of these young adults settled on territories (Piper et al. 2015). It is this sample of loons of known age and natal origin on which we based our analyses of NHPI and behavioral responses to human approach.

Observers visited lakes in the study area at the beginning of each breeding season to identify territorial pairs from leg bands. Thereafter, we made weekly hour-long observations at each active territory from canoes between 0500 and 1300 during the period 25 April to 7 August from 1992 to 2023. Once incubation behavior had been identified, observers visited all nests that had been active at the time of the last visit to confirm continued incubation. If incubation had ended, observers first searched for chicks and documented the number and approximate age of chicks based on their size. If no chicks were found, the nest was inspected and all shoreline area within 5 meters of the site was examined for eggshell fragments or other evidence with which to assess the cause of failure. For each lake included in the study, we also estimated pH using a pH meter (YSI Pro Plus; YSI Incorporated) using the mean of 2 readings taken in August 2005.

To measure risk assessment during incubation, an observer first recorded their distance from a loon in clear view on a nest at the beginning of an observation period. The observer then paddled the canoe slowly but directly towards the nest at a steady pace (ca. 1 m/s), and recorded the distance in meters at which the incubating loon flushed off the nest. Start distance was recorded as the distance of the observer in meters from the animal at the start of the approach and calculated by either estimating canoe lengths and converting to meters or using a range finder. Following the nest approach, we measured the distance between the incubator and the nest 5 min later to ensure nest attendance was virtually unaffected by these approaches. In 78% of cases, the bird had resumed incubation within 5 min. In all instances, one of the pair members was within 100 m of the nest 5 min later. If the loon did not flush despite the observer's canoe approaching to make gentle contact with the nest, it was recorded as a “sticker”. Of the nests that were approached in our study, 61% successfully hatched chicks, a higher percentage than the overall nest success in the study system of 48% between 1992 and 2023 (*N* = 2730).

### Measurement of dock density and shoreline development

We measured shoreline development in 2 ways. First, we examined the extent of developed land cover around each study lake. We obtained a 2024 land cover raster file from the National Land Cover Database (U. S. Geological Survey 2024) and a shapefile of Wisconsin waterbodies from the Wisconsin Department of Natural Resources (2022). We transformed both the land cover raster and waterbodies shapefile to the coordinate reference system “EPSG:7573” using the sf package (Version 1.0-19; Pebesma 2018; Pebesma and Bivand 2023) and terra package (Version 1.8-29; Hijmans et al. 2026), respectively. We only selected lakes that matched the waterbody identification codes of our study lakes. We used st_boundary (Pebesma 2018; Pebesma and Bivand 2023) to create a line feature around each lake and then used st_buffer (Pebesma 2018; Pebesma and Bivand 2023) to create 100-meter buffers around each line feature to quantify developed land cover in close proximity to lake shorelines. We converted the line features around each lake to polygons using st_polygonize (Pebesma 2018; Pebesma and Bivand 2023) and subtracted the lake polygon features from the buffer features using st_difference (Pebesma 2018; Pebesma and Bivand 2023) to only include the area of the buffers on land. We cropped and masked the land cover raster to the extent of the buffers using the crop function in the raster package (Version 3.6-31; Hijmans 2025). We extracted the number of cells of each land cover value for each buffer using the extract() function (Hijmans 2025) and summed the number of cells that were designated as developed land cover (Developed, Open Space; Developed, Low Intensity; Developed, Medium Intensity; and Developed, High Intensity). We summed the total number of cells of all land cover types for each buffer. We divided the sum of cells designated as developed land cover types by the total number of cells of all land cover types within each buffer to determine the proportion of developed land cover at each lake.

We used the density of docks as a second measure of shoreline development, reasoning that dock density might be a more precise indicator of human recreational activity. Dock density was equal to the number of docks on each territory, as determined from satellite images taken in 2023, divided by the length of shoreline abutting the lake, as reported by the Wisconsin Department of Natural Resources (2022). Dock density did not increase dramatically over the study period (Piper, personal observations). Dock density calculations were rounded to the nearest whole number. As an additional (binomial) measure of human activity, we quantified the presence or absence of a public boat ramp on the lake. While this is not a complete view of what is happening on a given lake, it provided a proxy for the amount of boat traffic. Lake size was extracted from data released by the Wisconsin Department of Natural Resources (2022).

### Statistical analysis

We addressed 2 primary questions regarding the impact of shoreline development on loon breeding behavior. First, we examined the extent of development on a loon's breeding lake relative to its natal lake in relation to NHPI, using developed land cover around each lake as our measure of shoreline development and dock density as a measure of human boating activity. Second, we looked at factors that influenced the FID, or distance at which incubating loons flushed from their nests, in response to nest approach, using both dock density and presence/absence of a public boat ramp as measures of human boating activity. All statistical analyses were conducted in R (Version 4.4.1; R Core Team 2024) using lme4 (Version 1.1-35.5; Bates et al. 2015), glmmTMB (Version 1.1.11; Brooks et al. 2017; McGillycuddy et al. 2025), lmerTest (Version 3.1-3; Kuznetsova et al. 2017) to determine *P*-values. Model assumptions were checked using the performance package (Version 0.13.0; Lüdecke et al. 2021) and DHARMa (Version 0.4.7; Hartig 2024).

### Natal habitat preference induction

To assess natal preference for shoreline development, we compiled data on all banded chicks that returned to the study area as breeding adults. A previous study on NHPI in loons examined 5 variables in the basic lake features classification and found that only lake size and pH were determinants in territory selection (Piper et al. 2013). Therefore, when we filtered our data, we included only instances in which lake size, pH, and dock density were available for the natal lake. We also included data on the distance between each individual's natal and breeding lakes, as loons exhibit short range natal dispersal. Given that a variety of habitat types were consistently vacant in the study area, it was not necessary to control for availability.

We fitted 2 linear regression models to examine the relationship between shoreline development on the breeding lake of identified individuals with shoreline development of their natal lake. Our analysis included individuals from 57 natal territories who settled on 61 breeding territories. Sampling was thinly distributed across lakes, with many lakes hosting a single bird (63% of natal lakes and 59% of breeding lakes). First, we fitted a linear mixed model using the R package lme4 (Bates et al. 2015) with breeding lake dock density as our dependent variable. We square-root transformed breeding lake dock density to meet the assumptions of normality. We included natal lake dock density, natal territory size, natal lake pH, breeding territory size, the distance in kilometers between natal and breeding lakes, and sex as fixed effects. We were specifically interested in whether natal lake dock density predicted breeding lake dock density in juveniles settling on territories for the first time. We added natal territory size, natal lake pH and distance between natal and breeding lake as candidate predictors to account for variance not associated with dock density but potentially driving settlement preference in loons. We included lake size to account for human bias of which lakes provide greater recreational activities. We included sex to account for sex-specific settlement choice, as males and females do not settle on territories as a pair and the arrival of a male does not necessarily predict the arrival of a female. We determined sex by either observing an individual's behavior when they intruded into or settled on territories, or through genetic analysis (Itoh 2001). We included natal territory as a random effect to control for nonindependence between individuals, as multiple individuals may share a natal territory. We checked model assumptions using diagnostic plots in the performance package (Version 0.13.0; Lüdecke et al. 2021). All assumptions were met.

To examine the relationship between developed land cover on the breeding lake of identified individuals with developed land cover of their natal lake, we fitted a generalized linear mixed model using the package glmmTMB (Version 1.1.11; Brooks et al. 2017) with the proportion of developed land on the breeding lake as our dependent variable. We included the proportion of developed land on the natal lake, natal territory size, natal lake pH, breeding territory size, the distance in kilometers between natal and breeding lakes, and sex as fixed effects. We included natal territory as a random effect. We used the beta family with a “logit” link function because our dependent variable was bounded on [0, 1) and we accounted for zero inflation in our dependent variable using “ziformula = ∼1”. All continuous variables were centered and scaled.

### Flight initiation distance

We collected FID with canoe approaches of incubating loons of known age. We calculated the number of days between when the observer approached and when the eggs hatched and removed all failed nest attempts. Since our dataset included a large number of stickers, we used a 2-step linear regression approach to assess what factors influenced both the propensity to flee and an individual's FID.

For FID, we removed all stickers prior to modeling. We fitted a linear mixed model with the following fixed effects: starting distance, dock density, boat ramp access, sex, and days before hatch. We included individual identity, territory, and year as random effects. We crossed our random effects because each individual could occupy more than 1 territory across years, and each territory hosted multiple individuals. Crossing accounts for the overlapping sources of nonindependence simultaneously. We log-transformed our response variable, FID, to meet our model's assumption of linearity. To determine whether disturbance influenced an individual's decision to flee their nest, we created a binomial dependent variable for stickers versus nonstickers. We fitted a generalized linear mixed model using the “binomial” family and “logit” link function with the “bobyqa” optimizer. We included starting distance, dock density, boat ramp access, sex, and days before hatch as fixed effects and individual identity, year and territory as random effects. All continuous variables were centered and scaled.

## Results

We analyzed the breeding lake settlement of 87 individuals between 1992 and 2023 whose first breeding lake was also in the study area and for whom we had data on lake attributes and shoreline development. Natal dock densities ranged from 0 to 16 with a mean of 6.34 ± 3.75 docks/km, and breeding dock densities ranged from 0 to 18 with a mean of 5.69 ± 4.39 docks/km. We first examined all study lakes to learn if occupancy of territories was correlated with dock density within them. We found no relationship between dock density and proportion of years that a lake was occupied (*r*^2^ = 0.006, *N* = 171 lakes, *P* = 0.31, 2-tailed correlation). Thus, we found no evidence that loons settled preferentially on lakes with few docks.

In our model testing the relationship between natal and breeding dock densities, we found a significant positive relationship between natal and breeding dock densities (estimate = 0.071; *P* = 0.043; Table 1 and Fig. 1); loons that were born on lakes with high dock densities also settled on lakes with high dock densities. There was a potential, but ultimately nonsignificant and weak, relationship between breeding territory size and breeding lake dock density (estimate = 0.001; *P* = 0.079). No other variables explained any significant variation in breeding territory dock density.

**Figure 1:**
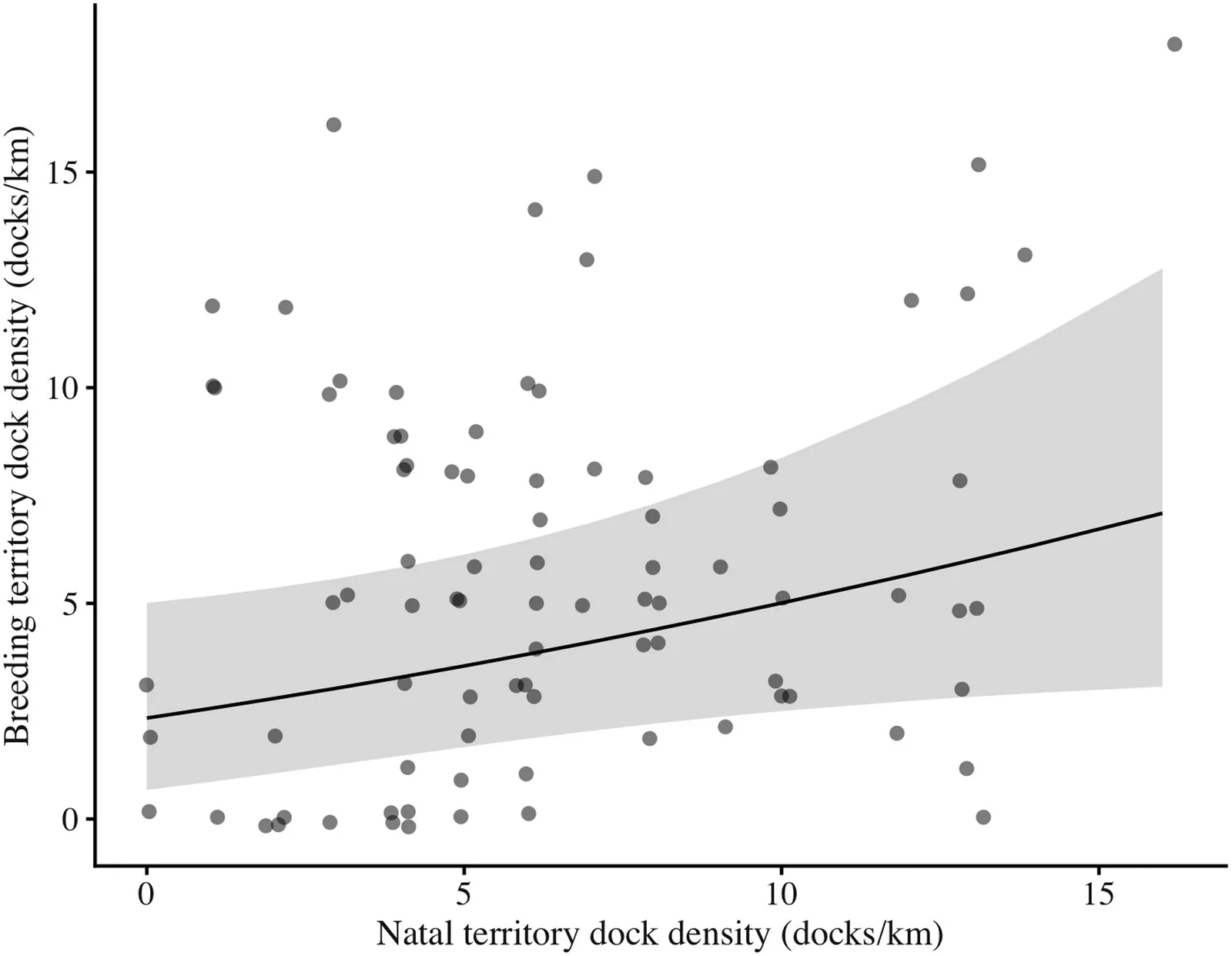
The relationship between natal territory dock density and breeding territory dock density.

**Table 1:** Results of the linear mixed model showing the relationship between natal dock density and breeding dock density.

Fixed effects	Estimate	SE	*P*
**Natal dock density**	**0**.**071**	**0**.**034**	**0**.**043**
Natal territory pH	−0.029	0.019	0.127
Natal territory size	<0.001	0.002	0.799
Distance between natal and breeding lakes	0.028	0.023	0.228
Breeding territory size	0.001	0.001	0.079
Sex (Male)	0.203	0.340	0.551
**Random Effect**	**Variance**	**SD**	
Natal territory	0.153	0.392	

Our model contained 87 unique observations. Reference classes include “Female” for sex. Bolded results indicate significant effects.

The proportion of shoreline covered by impervious surface on natal territories ranged from 0 to 0.445 with a mean of 0.128 ± 0.079 and the proportion of impervious surface cover on breeding territories ranged from 0 to 0.555 with a mean of 0.127 ± 0.112. We found no significant relationship between natal territory impervious surface cover and breeding territory impervious surface cover (Table 2). No other variables explained significant variation in breeding territory impervious surface cover.

**Table 2:** Results of the generalized linear mixed model showing the relationship between natal lake developed land cover and breeding lake developed land cover.

Fixed effects	Estimate	SE	*P*
Natal lake proportion developed land cover	0.024	0.107	0.822
Natal territory pH	0.139	0.717	0.846
Natal territory size	0.089	0.104	0.394
Distance between natal and breeding lakes	0.158	0.105	0.132
Breeding territory size	0.066	0.082	0.424
Sex (Male)	0.341	0.252	0.177
**Random Effect**	**Variance**	**SD**	
Natal territory	<0.001	<0.001	

Our model contained 87 unique observations. Reference classes include “Female” for sex. All continuous variables were centered and scaled.

We analyzed 76 unique FID measurements of 41 individuals. Sixteen individuals were sampled across multiple years, with an average of 1.56 approaches per individual and a minimum and maximum total number of approaches being 1 and 5, respectively. Mean FID for females was 9.98 m ± 15.2 m and mean FID for males was 7.86 ± 12.7 m. We found no association between either dock density (*P* = 0.482; Table 3) or boat ramp access (*P* = 0.744; Table 3) and FID. Starting distance had a significant positive association with FID (estimate = 0.010; *P* = 0.009). Sex and days before hatch did not explain significant variation in FID.

**Table 3:** Results of the generalized linear mixed model of FID, with 76 unique observations of 41 individuals across 16 yr.

Fixed effects	Estimate	SE	*P*
**Starting Distance**	**0**.**010**	**0**.**004**	**0**.**009**
Dock Density	0.012	0.017	0.482
Boat Ramp Access (Yes)	−0.060	0.183	0.744
Sex (Male)	−0.262	0.156	0.101
Days Before Hatch	0.006	0.005	0.286
**Random Effects**	**Variance**	**SD**	
Loon ID	0.092	0.303	
Territory	0.000	0.000	
Year	0.037	0.193	

Reference classes include “No” for boat ramp access and “Female” for sex. Bolded results indicate significant effects.

We fitted a generalized linear mixed model of the propensity to leave the nest using 161 observations of 69 individuals. Neither dock density (*P* = 0.945; Table 4) nor boat ramp access (*P* = 0.318; Table 4) significantly explained variation in an individual's propensity to flee the nest. The closer eggs were to hatching, the more likely loons were to remain on the nest during approach (estimate = −0.917; *P* = 0.022; Fig. 2). Sex approached significance, with males slightly more likely to flush (estimate = 2.35; *P* = 0.056).

**Figure 2:**
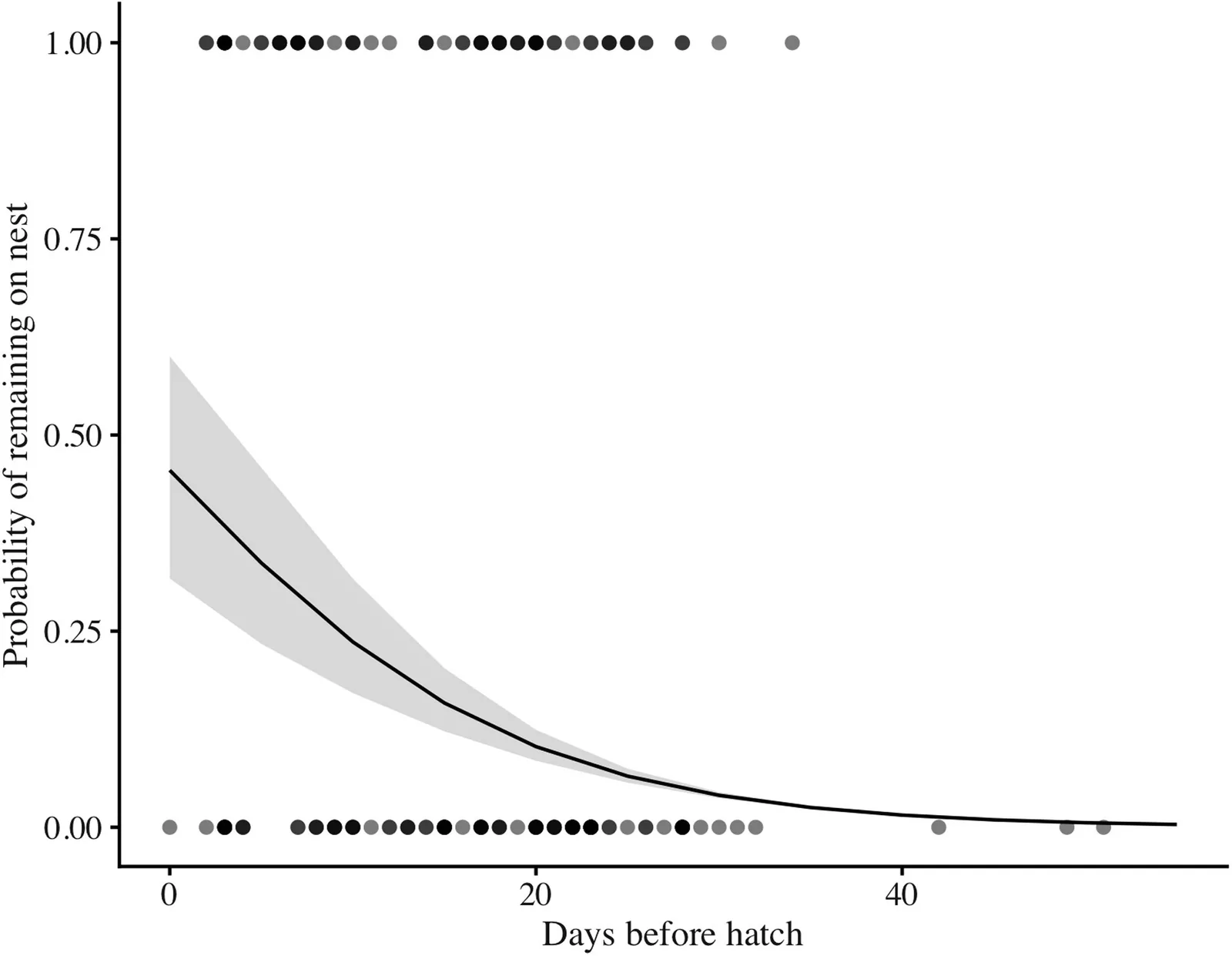
The relationship between whether an individual remained on nest and days before hatch.

**Table 4:** Results of the generalized linear mixed model of the propensity to flee, with 161 unique observations of 69 individuals across 16 yr.

Fixed effects	Estimate	SE	*P*
Starting Distance	0.391	0.648	0.546
Dock Density	0.031	0.454	0.945
Boat Ramp Access (Yes)	−1.12	1.12	0.318
Sex (Male)	2.35	1.23	0.056
**Days Before Hatch**	**−0**.**917**	**0**.**400**	**0**.**022**
**Random Effects**	**Variance**	**SD**	
Loon ID	6.02	2.45	
Territory	0.294	0.542	
Year	0.265	0.515	

Reference classes include “No” for boat ramp access and “Female” for sex. All continuous variables were centered and scaled. Bolded results indicate significant effects.

## Discussion

We detected 2 clear patterns in nest defense by loons. In defending their nest and young, animals are expected to balance the value of their current breeding effort against their own residual reproductive value (Pianka and Parker 1975). As long-lived animals with high renesting potential, loons would not be expected to assume great risk in defending any single breeding attempt. Consistent with this expectation, loons usually flushed from nests when observers were at least a meter away, such that the risk to their lives was minimal.

A second pattern was temporal. That is, although nest defense was rather limited overall, defense varied according to stage of incubation. Specifically, loons were more likely to remain on the nest when close to the hatch date. This finding is consistent with parental investment theory, which predicts that animals should increase the extent of their nest defense as the value of the offspring increases (Montgomerie and Weatherhead 1988). An increase in parental defense of young and nests has been reported in numerous other avian species (eg, Brunton 1990; Sieder et al. 2025; Lawson et al. 2026).

Interestingly, although individuals differed greatly in degree of nest defense, those on highly developed lakes showed no more nest defense than those on remote lakes. This finding was counter to our prediction and to the findings of Ruggles (1994). Evidently, loons nesting on busy lakes do not come to tolerate human activity, nor does the set of loons breeding on busy lakes differ from loons on remote lakes in terms of nest defense. Rather, our findings suggest ontogenetic and temporal factors may be more important to loon risk-assessment than habitat characteristics. Our results add to a growing body of work which has shown that variation in nest defense based on habitat features is species-specific and based in part on life history traits (Knight et al. 1987; Kunca and Yosef 2016; Merrill et al. 2016).

More consequential was our finding that loons selected breeding lakes with a dock density similar to their natal lake, a pattern that provides evidence for NHPI on the basis of an anthropogenic factor. Other studies in mammals and birds have shown both positive and negative NHPI (ie a tendency to favor or avoid attributes of the natal site) (Milleret et al. 2019; Orgeret et al. 2024). For example, Milleret et al. (2019) found that female wolves (*Canis lupus*) reared in proximity to humans tended to settle far from human settlements, whereas red kites (*Milvus milvus*) exhibited both preference and avoidance of human-altered natal habitats depending on life history stage (Orgeret et al. 2024). In short, human alterations of vertebrates' natal habitats have been shown to affect NHPI in complex ways.

Shoreline development is a frequent and important indicator of anthropogenic impacts on animals that use aquatic and riparian habitats (Racey and Euler 1983; Traut and Hostetler 2003; Donaldson et al. 2007). Indeed, impacts on natural patterns of vegetation, drainage, and littoral aquatic communities make shoreline development significant for many animal species (Chhor et al. 2020). However, our finding that loons cued on dock density, rather than shoreline development, suggests that loons focus less on lake shorelines or littoral habitats during habitat imprinting and settlement and instead on human boating and angling activity offshore. This interpretation is consistent with loons' tendency to confine most of their activity and foraging to the pelagic zone, not the littoral region of their lakes.

Although the relevance of dock density to loon biology is logical in a broad sense, the specific preference of loons for lakes with a similar density of docks to their natal one does not have an obvious explanation. We suggest that dock density might be an indicator of the abundance and/or the species composition of small fishes present. For example, dock density in Minnesota lakes has been found recently to be positively associated with richness of fish species (Dustin and Vondracek 2017). Therefore, it is possible that loons reared on lakes with a wide or narrow range of fish species simply prefer to settle as adults on lakes that also support a diversity or paucity of fish species. Alternatively, dock density might lead to a species composition of prey fishes in a more specific way. Overfishing of walleye (*Sander vitreus*), the chief gamefish in our study area, has been shown to increase abundance and biomass of their prey species: white sucker (*Catostomus commersonii*), cisco (*Coregonus artedi*), and yellow perch (*Perca flavescens*) (Thao et al. 2016). Thus, lakes with high dock density might contain a high abundance of species favored by walleye, whereas those with few docks contain low numbers of such prey. If so, loons might learn to favor a certain walleye-dependent assemblage of prey species when young and retain that preference during territory settlement. Consistent with this idea, past work in this system found that while higher dock density negatively affected adult male mass, chicks were significantly heavier when dock density was higher, a pattern potentially explained by increased recreational pressure promoting an abundance of smaller fishes beneficial for chick feeding (Piper et al. 2024).

Our finding that loons from high-activity lakes choose high-activity lakes as breeding habitat suggests that development does shape behavior at the settlement stage, which may have important implications for loon conservation. Loon populations are declining at the southern edge of their breeding range because of climate change and human development, which has resulted in a reduced survival of young adults and lower productivity (Piper et al. 2020; Saunders et al. 2021). Presuming that human activity can reach some threshold beyond which it negatively impacts loons, growing preference of loons for highly developed lakes because of the steady increase in vacation homes on northern shorelines might produce a harmful ecological trap that could lead to further population decline.

This has relevance to habitat selection well beyond the loon system. The NHPI we found suggests that animals establish familiarity with the benefits and drawbacks of living in a human-altered habitat and attempt to choose a similar habitat when they settle as an adult. Indeed, multiple ungulate species have been observed to imprint on certain human-altered habitats early in life and settle in similar ones as adults (Larue et al. 2018; Hooven et al. 2023). Furthermore, caracals (*Caracal caracal*) will flexibly select habitat in response to anthropogenic disturbance, with individuals increasing their selection for proximity to urban areas as the proportion of urban areas increases (Serieys et al. 2023). Whether NHPI on human-altered habitats is adaptive or not is a separate question. We know of no study to date that has measured the fitness impact of such behavior.

There is evidence that NHPI is more widespread than currently reported (Piper et al. 2013; Merrick and Koprowski 2016), and these habitat selection cues have important implications for understanding species distribution. Past studies have shown that habitat settlement driven by NHPI shapes how populations are distributed across the landscape (Fletcher et al. 2015). Human alteration of landscapes can have a legacy effect on species distribution by drastically altering breeding habitats in many species, and therefore unintentionally increasing animals' preferences for altered habitats through NHPI. Long-term studies of individually marked animals are particularly valuable for improving our understanding of settlement behavior in the face of environmental change. Habitats chosen by an animal also influence fitness outcomes, as the quality of habitat influences the benefits received by that individual as well as the risk of predation or nest disturbance. Understanding whether there is a threshold for withstanding anthropogenic pressures before changing settlement behaviors, or whether juvenile animals who experience a high degree of adversity on their natal grounds do not survive the juvenile stage to return as breeders, may reveal key limits to a species' adaptability in a rapidly changing environment.

Our results show some plasticity in loon habitat selection in response to human activity. However, loons in many regions are producing fewer offspring, and first-time breeders are returning to their breeding grounds in shrinking numbers. Loon populations are declining, especially at the southern edge of their breeding range, because of climate change and human development (Saunders et al. 2021). These findings together suggest that early-life habitat selection may interact with climate and development pressures to accelerate loon population declines.

As human encroachment into northern regions intensifies, ecologists are increasingly tasked with maintaining populations of animals that have not had to cope with human activity in the past. The habitat selection patterns that we have observed are a conspicuous example of the challenges that animals face in modifying their behavior in response to anthropogenic disturbance. On the one hand, our findings show that loons have some behavioral flexibility in responding to human disturbance, as individuals reared on lakes with higher human interaction choose to settle on similar lakes as adult breeders. On the other hand, animals' tolerance for humans might cause them to settle in habitats where young is increasingly hard to rear, owing to negative anthropogenic impacts.

## Data Availability

Analyses reported in this article can be reproduced using the data provided by Renkey et al. (2026). The datasets and code generated during and/or analyzed for the study are available in OSF here: https://osf.io/6fncb/.

## References

[arag133-B1] Bates D, Mächler M, Bolker B, Walker S. 2015. Fitting linear mixed-effects models using lme4. J Stat Softw. 67:1–48. 10.18637/jss.v067.i01.

[arag133-B2] Brooks ME et al 2017. glmmTMB balances speed and flexibility among packages for zero-inflated generalized linear mixed modeling. R J. 9:378–400. 10.32614/RJ-2017-066.

[arag133-B3] Brunton DH . 1990. The effects of nesting stage, sex, and type of predator on parental defense by killdeer (*Charadrius vociferous*): Testing models of avian parental defense. Behav Ecol Sociobiol. 26:181–190. 10.1007/BF00172085.

[arag133-B4] Burgess NM, Meyer MW. 2008. Methylmercury exposure associated with reduced productivity in common loons. Ecotoxicology. 17:83–91. 10.1007/s10646-007-0167-8. 18038272

[arag133-B5] Caron JA, Robinson WL. 1994. Responses of breeding common loons to human activity in upper Michigan. Hydrobiologia. 279–280:431–438. 10.1007/BF00027874.

[arag133-B6] Chhor AD, Glassman DM, Smol JP, Vermaire JC, Cooke SJ. 2020. Ecological consequences of shoreline armoring on littoral fish and benthic macroinvertebrate communities in an eastern Ontario lake. Aquat Sci. 82:73. 10.1007/s00027-020-00740-0.

[arag133-B7] Cooper WE Jr, Blumstein DT. 2015. Escaping from predators: an integrative view of escape decisions. Cambridge University Press.

[arag133-B8] Davis JM, Stamps JA. 2004. The effect of natal experience on habitat preferences. Trends Ecol Evol. 19:411–416. 10.1016/j.tree.2004.04.006. 16701298

[arag133-B9] Donaldson MR, Henein KM, Runtz MW. 2007. Assessing the effect of developed habitat on waterbird behaviour in an urban riparian system in Ottawa, Canada. Urban Ecosyst. 10:139–151. 10.1007/s11252-006-0015-2.

[arag133-B10] Dustin DL, Vondracek B. 2017. Nearshore habitat and fish assemblages along a gradient of shoreline development. N Am J Fish Manag. 37:432–444. 10.1080/02755947.2017.1280567.

[arag133-B11] Evers DC . 1993. A replicable capture method for adult and juvenile common loons on their nesting lakes. In: Stockwell F, editors. In proceedings from the 1992 conference on the loon and its ecosystem: status, management and environmental concerns. North American Loon Fund. p. 214–220.

[arag133-B12] Evers DC . 2004. Status assessment and conservation plan for the Common Loon (Gavia immer) in North America. U.S. Fish and Wildlife Service, Hadley, Massachusetts.

[arag133-B13] Fletcher RJ et al 2015. Affinity for natal environments by dispersers impacts reproduction and explains geographical structure of a highly mobile bird. Proc R Soc Lond B Biol Sci. 282:20151545. 10.1098/rspb.2015.1545. PMC4571708 26336178

[arag133-B14] Grade TJ, Pokras MA, Laflamme EM, Vogel HS. 2018. Population-level effects of lead fishing tackle on common loons. J Wildl Manage. 82:155–164. 10.1002/jwmg.21348.

[arag133-B15] Hartig F . 2024. DHARMa: Residual diagnostics for hierarchical (multi-level/mixed regression) models. R package version 0.4.7. [updated 2026 Jan 26]. https://CRAN.R-project.org/package=DHARMa.

[arag133-B16] Hijmans R, Brown A, Barbosa AM, Cordano E, Dyba K. 2026. terra: Spatial Data Analysis. R package version 1.9-51. [updated 2026 Jan 26]. https://github.com/rspatial/terra.

[arag133-B17] Hijmans RJ . 2025. raster: Geographic data analysis and modeling. R package version 3.6-31. [updated 2026 Jan 26]. https://cran.r-project.org/web/packages/raster/index.html

[arag133-B18] Hooven ND, Springer MT, Nielsen CK, Schauber EM. 2023. Influence of natal habitat preference on habitat selection during extra-home range movements in a large ungulate. Ecol Evol. 13:e9794. 10.1002/ece3.9794. PMC9897958 36760707

[arag133-B19] Itoh Y . 2001. Identification of the sex of a wide range of carinatae birds by PCR using primer sets selected from chicken EE0.6 and its related sequences. J Hered. 92:315–321. 10.1093/jhered/92.4.315. 11535643

[arag133-B20] Knight RL, Grout DJ, Temple SA. 1987. Nest-defense behavior of the American crow in urban and rural areas. Condor. 89:175. 10.2307/1368772.

[arag133-B21] Kunca T, Yosef R. 2016. Differential nest-defense to perceived danger in urban and rural areas by female Eurasian sparrowhawk (*Accipiter nisus*). PeerJ. 4:e2070. 10.7717/peerj.2070. PMC4941787 27441105

[arag133-B22] Kuznetsova A, Brockhoff PB, Christensen RHB. 2017. lmerTest package: tests in linear mixed effects models. J Stat Softw. 82. 10.18637/jss.v082.i13.

[arag133-B23] Larue B, Côté SD, St-Laurent M, Dussault C, Leblond M. 2018. Natal habitat preference induction in large mammals—like mother, like child? Ecol Evol. 8:12629–12640. 10.1002/ece3.4685. PMC6309006 30619569

[arag133-B24] Lawson SL, Zbaracki AH, Campbell RML, Fraser EM, Kikuchi DW. 2026. Red-winged blackbirds adjust aggression with brood value and predator type, but show broadly similar responses to snake predators of differing risk. Behav Ecol. 37:arag089. 10.1093/beheco/arag089.

[arag133-B25] Lawson ZJ, Gaeta JW, Carpenter SR. 2011. Coarse woody habitat, lakeshore residential development, and largemouth bass nesting behavior. N Am J Fish Manag. 31:666–670. 10.1080/02755947.2011.608990.

[arag133-B26] Li C, Monclús R, Maul TL, Jiang Z, Blumstein DT. 2011. Quantifying human disturbance on antipredator behavior and flush initiation distance in yellow-bellied marmots. Appl Anim Behav Sci. 129:146–152. 10.1016/j.applanim.2010.11.013.

[arag133-B27] Lindsay AR, Gillum SS, Meyer MW. 2002. Influence of lakeshore development on breeding bird communities in a mixed northern forest. Biol Conserv. 107:1–11. 10.1016/S0006-3207(01)00260-9.

[arag133-B28] Livezey KB, Fernández-Juricic E, Blumstein DT. 2016. Database of bird flight initiation distances to assist in estimating effects from human disturbance and delineating buffer areas. J Fish Wildlife Manage. 7:181–191. 10.3996/082015-JFWM-078.

[arag133-B29] Lüdecke D, Ben-Shachar MS, Patil I, Waggoner P, Makowski D. 2021. Performance: an R package for assessment, comparison and testing of statistical models. J Open Source Softw. 6:3139. 10.21105/joss.03139.

[arag133-B30] McGillycuddy M, Warton DI, Popovic G, Bolker BM. 2025. Parsimoniously fitting large multivariate random effects in glmmTMB. J Stat Softw. 112:1–19. 10.18637/jss.v112.i01.

[arag133-B31] McGowan MM, Patel PD, Stroh JD, Blumstein DT. 2014. The effect of human presence and human activity on risk assessment and flight initiation distance in skinks. Ethology. 120:1081–1089. 10.1111/eth.12281.

[arag133-B32] Merrick MJ, Koprowski JL. 2016. Evidence of natal habitat preference induction within one habitat type. Proc R Soc Lond B Biol Sci. 283:20162106. 10.1098/rspb.2016.2106. PMC5124104 27807266

[arag133-B33] Merrill L, Baehl EM, Ripple KE, Benson TJ. 2016. Do birds alter levels of parental investment based on nest-site habitat features? Ethology. 122:859–868. 10.1111/eth.12535.

[arag133-B34] Milleret C et al 2019. Testing the influence of habitat experienced during the natal phase on habitat selection later in life in Scandinavian wolves. Sci Rep. 9:6526. 10.1038/s41598-019-42835-1. PMC6484024 31024020

[arag133-B35] Møller AP . 2015. The value of a mouthful: flight initiation distance as an opportunity cost. Euro J Ecol. 1:43–51. 10.1515/eje-2015-0006.

[arag133-B36] Montgomerie RD, Weatherhead PJ. 1988. Risks and rewards of nest defence by parent birds. Q Rev Biol. 63:167–187. 10.1086/415838.

[arag133-B37] Orgeret F et al 2024. Imprinted habitat selection varies across dispersal phases in a raptor species. Sci Rep. 14:26656. 10.1038/s41598-024-75815-1. PMC11535207 39496617

[arag133-B38] Paruk JD et al 2021. Common Loon (Gavia immer), version 2.0. In: Rodewald PG, Keeney BK, editors. Birds of the world. Cornell Lab of Ornithology.

[arag133-B39] Pebesma E . 2018. Simple features for R: standardized support for spatial vector data. R J. 10:439–446. 10.32614/RJ-2018-009.

[arag133-B40] Pebesma E, Bivand R. 2023. Spatial data science: with applications in R. Chapman and Hall/CRC.

[arag133-B41] Perales KM, Vander Zanden MJ. 2023. Lakeshore residential development as a driver of aquatic habitat and littoral fish communities: a cross-system study. Ecol Appl. 33:e2896. 10.1002/eap.2896. 37305904

[arag133-B42] Pianka ER, Parker WS. 1975. Age-specific reproductive tactics. Am Nat. 109:453–464. 10.1086/283013.

[arag133-B43] Piper WH et al 2006. Prospecting in a solitary breeder: chick production elicits territorial intrusions in common loons. Behav Ecol. 17:881–888. 10.1093/beheco/arl021.

[arag133-B44] Piper WH et al 2015. Territory settlement in common loons: no footholds but age and assessment are important. Anim Behav. 104:155–163. 10.1016/j.anbehav.2015.03.013.

[arag133-B45] Piper WH, Glines MR, Rose KC. 2024. Climate change-associated declines in water clarity impair feeding by common loons. Ecology. 105:e4291. 10.1002/ecy.4291. 38556944

[arag133-B46] Piper WH, Grear J, Hoover B, Lomery E, Grenzer LM. 2020. Plunging floater survival causes cryptic population decline in the Common Loon. Condor. 122:duaa044. 10.1093/condor/duaa044. PMC11363150 39220437

[arag133-B47] Piper WH, Grear JS, Meyer MW. 2012. Juvenile survival in common loons *Gavia immer*: effects of natal lake size and pH. J Avian Biol. 43:280–288. 10.1111/j.1600-048X.2012.05633.x.

[arag133-B48] Piper WH, Palmer MW, Banfield N, Meyer MW. 2013. Can settlement in natal-like habitat explain maladaptive habitat selection? Proc R Soc Lond B Biol Sci. 280:20130979. 10.1098/rspb.2013.0979. PMC3712445 23804619

[arag133-B49] Racey GD, Euler DL. 1983. Changes in mink habitat and food selection as influenced by cottage development in central Ontario. J Appl Ecol. 20:387. 10.2307/2403515.

[arag133-B50] R Core Team . 2024. R: A language and environment for statistical computing. R Foundation for Statistical Computing. [updated 2026 Jan 20]. https://www.R-project.org/.

[arag133-B51] Renkey E, Adler KA, Blumstein DT, Piper WH. 2026. Data from: Shoreline development influences common loon territory selection but not extent of nest defense. OSF. https://osf.io/6fncb/overview?view_only=9c66a7819e914eda96847182864ac357

[arag133-B52] Ruggles AK . 1994. Habitat selection by loons in southcentral Alaska. Hydrobiologia. 280:421–430. 10.1007/BF00027873.

[arag133-B53] Saunders SP et al 2021. Interrelated impacts of climate and land-use change on a widespread waterbird. J Anim Ecol. 90:1165–1176. 10.1111/1365-2656.13444. 33754380

[arag133-B54] Schindler DE, Geib SI, Williams MR. 2000. Patterns of fish growth along a residential development gradient in north temperate lakes. Ecosystems. 3:229–237. 10.1007/s100210000022.

[arag133-B55] Serieys LEK et al 2023. Anthropogenic activities and age class mediate carnivore habitat selection in a humandominated landscape. iScience. 26:107050. 10.1016/j.isci.2023.107050. PMC10391726 37534145

[arag133-B56] Sieder S et al 2025. Food availability affects parental anti-predator behaviour in red kites. J Avian Biol. 2025:e03475. 10.1002/jav.03475.

[arag133-B57] Spilman CA, Schoch N, Porter WF, Glennon MJ. 2014. The effects of lakeshore development on common loon (*Gavia immer*) productivity in the Adirondack park, New York, USA. Waterbirds. 37(sp1):94–101. 10.1675/063.037.sp112.

[arag133-B58] Thao C, Mosindy TE, Venturelli PA. 2016. Fish community changes in Shoal Lake, Canada, following the overexploitation of a top predator. J Freshwater Ecol. 31:443–450. 10.1080/02705060.2016.1173120.

[arag133-B59] Traut AH, Hostetler ME. 2003. Urban lakes and waterbirds: effects of development on avian behavior. Waterbirds. 26:290–302. 10.1675/1524-4695(2003)026[0290:ULAWEO]2.0.CO;2.

[arag133-B60] Twardochleb LA, Olden JD. 2016. Human development modifies the functional composition of lake littoral invertebrate communities. Hydrobiologia. 775:167–184. 10.1007/s10750-016-2727-5.

[arag133-B61] Uchida K, Blumstein DT. 2021. Habituation or sensitization? Long-term responses of yellow bellied marmots to human disturbance. Behav Ecol. 32:668–678. 10.1093/beheco/arab016.

[arag133-B62] U.S. Geological Survey (USGS). 2024. Annual NLCD Collection 1 science products (ver. 1.1, June 2025) [Data release]. U.S. Geological Survey. 10.5066/P94UXNTS.

[arag133-B63] Weatherhead PJ . 1979. Do savannah sparrows commit the concorde fallacy? Behav Ecol Sociobiol. 5:373–381. 10.1007/BF00292525.

[arag133-B64] Wisconsin Department of Natural Resources . 2022. 24K Hydro Waterbodies. [updated 2026 Jan 26]. https://data-wi-dnr.opendata.arcgis.com/datasets/31f1f67253074ef9afe46cd905bff07a_1/explore?location=45.760340%2C-89.370858%2C12

[arag133-B65] Woodford JE, Meyer MW. 2003. Impact of lakeshore development on green frog abundance. Biol Conserv. 110:277–284. 10.1016/S0006-3207(02)00230-6.

[arag133-B66] Ydenberg RC, Dill LM. 1986. The economics of fleeing from predators. Adv Study Behav. 16:229–249. 10.1016/S0065-3454(08)60192-8.

